# Laser Scanning on Road Pavements: A New Approach for Characterizing Surface Texture

**DOI:** 10.3390/s120709110

**Published:** 2012-07-03

**Authors:** Gabriele Bitelli, Andrea Simone, Fabrizio Girardi, Claudio Lantieri

**Affiliations:** DICAM, Department of Civil, Environmental and Materials Engineering, University of Bologna, Viale del Risorgimento 2, 40136 Bologna, Italy; E-Mails: andrea.simone@unibo.it (A.S.); fabrizio.girardi@unibo.it (F.G.); claudio.lantieri2@unibo.it (C.L.)

**Keywords:** road pavements, texture, asphalt concrete, laser scanner, 3D

## Abstract

The surface layer of road pavement has a particular importance in relation to the satisfaction of the primary demands of locomotion, such as security and eco-compatibility. Among those pavement surface characteristics, the “texture” appears to be one of the most interesting with regard to the attainment of skid resistance. Specifications and regulations, providing a wide range of functional indicators, act as guidelines to satisfy the performance requirements. This paper describes an experiment on the use of laser scanner techniques on various types of asphalt for texture characterization. The use of high precision laser scanners, such as the triangulation types, is proposed to expand the analysis of road pavement from the commonly and currently used two-dimensional method to a three-dimensional one, with the aim of extending the range of the most important parameters for these kinds of applications. Laser scanners can be used in an innovative way to obtain information on areal surface layer through a single measurement, with data homogeneity and representativeness. The described experience highlights how the laser scanner is used for both laboratory experiments and tests *in situ*, with a particular attention paid to factors that could potentially affect the survey.

## 1. Introduction

**Figure 1.**
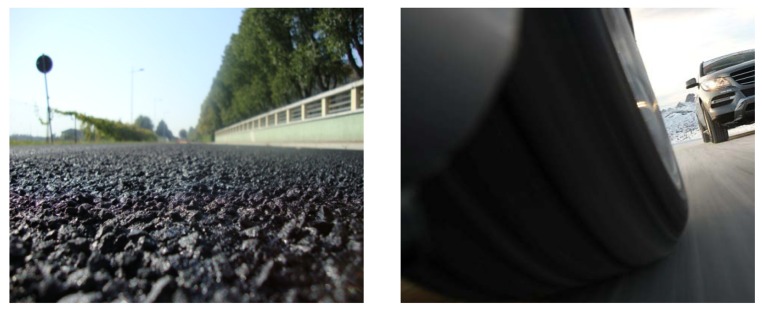
Pavement texture and tire-road contact.

It is well known that the characteristics of the road surface, especially for the surface layer, are essential to satisfy some primary requests, such as user safety and noise pollution. Road performances are being evaluated and classified by the adoption of certain parameters that affect various characteristics of the road, one of which is the surface texture; it is strictly related also to the tire-road contact (Figure 1).

**Figure 2.**
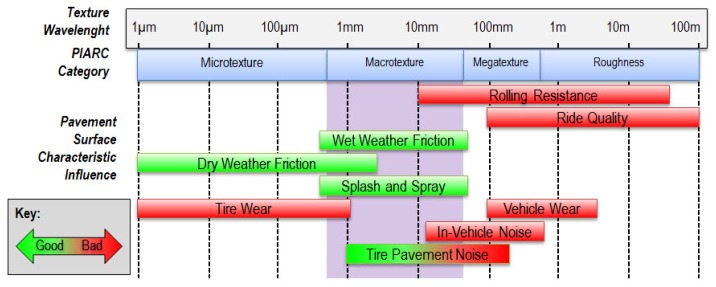
Relationship between texture and characteristics of the pavement surface [1,2].

The texture of a pavement is defined as “the deviation of a pavement surface from a true planar surface”. To give an idea of representation of the texture, if we section the pavement surface by means of a vertical plane, it is possible to extract a profile. The texture is a component that can be subject to different scales of investigation, where the discriminant is represented by the wavelength. The wavelength is defined as “the minimum distance between periodical repeated parts of the curves” (ISO 13473-1). Figure 2 illustrates the identified intervals, which correspond to different aspects influencing the locomotion.

The main performance characteristics of the top layer surface depends on the texture: skid resistance, ride quality, drainage, rolling resistance, tire-pavement noise and traffic vibration [3].

The two levels of texture that predominantly affect friction are micro-texture and macro-texture [4]. Micro-texture is mainly responsible for pavement friction at low speeds and in dry condition, whereas macro-texture is mainly responsible for reducing the potential for separation of tire and pavement surface due to hydroplaning, and for inducing friction caused by hysteresis for vehicles traveling at high speeds.

The micro-texture, which corresponds to wavelengths less than 0.5 mm and peak-to-peak amplitudes of the profile ranging between 1 micron and 0.2 mm, is related to the roughness of the individual stone elements used in the surface layer and to the natural mineral aggregate.

The macro-texture, which corresponds to wavelengths between 0.5 mm and 50 mm, with peak-to-peak amplitudes between 0.2 mm and 10 mm, is related instead to all the inter-granular roughness, and therefore essentially depends on the mixture composition (grading curve) and the mode of paving. This range is the focus of attention of the presented work.

The macro-texture of the pavement plays a fundamental role in the drainage and the vehicle handling at high speed [5]. The drainage capacity is particularly important, because two factors rely on it: the rate of rain that runoff from the pavement and the water film present on the surface when it rains [6].

It is clear, then, that the knowledge of the roughness, and the possibility to measure it, is particularly important for the safety of road users [7].

Many of the techniques currently used to obtain texture indicators produce two-dimensional values, associated to single profiles; this approach, adopted by current rules, has the disadvantage of a low representativeness of the measurement and the necessity of various tests to obtain a representative average value of the indexes. This technique does not allow investigations with additional indicators that derive from a three-dimensional analysis [8,9]. For this purpose, an experiment using a laser scanning system, in particular a triangulation one, was conducted. Several tests were carried out on differently characterized samples in order to develop methodologies useful for laboratory and *in situ* monitoring; they provide indices not employed in road engineering at the present time.

## 2. Instrument and Working Principles

In the last several years, a wide range of tools for 3D digitizing came onto the market. These instruments are used in a variety of applications in various fields (medicine, mechanical engineering, civil, cultural heritage, forensics, *etc.*) and allow the acquisition of objects in a wide range of sizes and on different scales [10]. Two basic approaches can be distinguished: image-based systems, where 3D models are generated using digital images, and range-based systems that use scanning devices with different technologies [11].

For the subsequent experiments this paper refers to a triangulating desktop laser scanner. Triangulation lasers devices, thanks to the continuous progress made on electronics and sensors in the last years, are proven as a reliable technology for non-contact surface measurements. The laser triangulation is an active stereoscopic technique that, based on the principle of the topographic forward intersection, is able to determine the position of a point in the space defined by the instrumental reference system.

**Figure 3.**
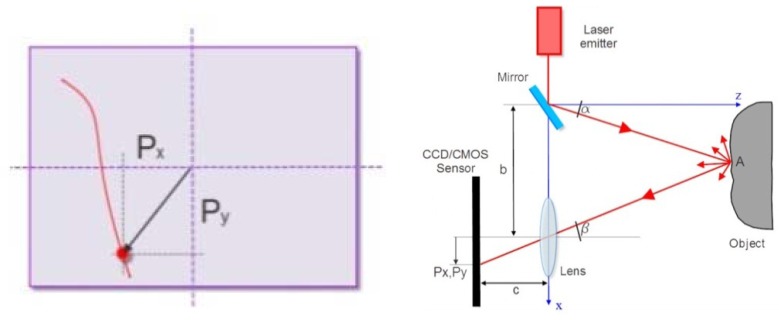
Triangulation laser scanner outline.

According to the outline in Figure 3, the laser emitter produces a beam of energy that comes from the instrument at an angle (α), which is known due to a prior calibration of the rotating mirror. It hits the surface of the object at the point (A) that is being measured. The laser beam undergoes a reflection, where the magnitude of the reflection depends on the type of surface targeted; a part of the reflected signal hits the receiving sensor (usually a CCD or CMOS) positioned at a known distance, called the baseline (b), from the emitter. The angle (β) of the incoming ray is unknown, but it is possible to calculate it by applying the trigonometric formulas (1), and through the knowledge of the focal length (c) and the position of the laser spot (Px, Py) recorded by the sensor array. By repeating this operation for all the points in which it is possible to discretize the surface of the object, it is possible to determine their coordinates according to the relations (2), and then discretize the surface through a three-dimensional point cloud.

(1) $$\begin{array}{cc} \tan\beta =\frac{P_{x}}{c}\;; & \tan\gamma =\frac{P_{y}}{c} \end{array}$$

(2) $$\begin{array}{ccc} x_{A}=\frac{b}{1+\frac{\tan\beta }{\tan\alpha }}\;; & y_{A}=\frac{b}{\frac{\tan\alpha }{\tan\gamma }+\frac{\tan\beta }{\tan\gamma }}\;; & z_{A}=\frac{b}{\tan\alpha +\tan\beta } \end{array}$$

Following a process of triangulation, the point clouds can be converted into a mesh of triangles, which constitute the 3D surface of the object. According to the technology adopted by the manufacturing tools and the type of footprint projected on the object's surface, the lasers can be classified as “single spot”, “line” or “multi-line” systems.

One acquisition is generally not sufficient to recover the entire surface for freeform objects. For this reason, in order to describe the surface in the best way, other scan positions from different point of view are adopted.

The laser scanner used in this work is produced by Next Engine Inc. and is based on the Multi-stripe Laser Triangulation (MLT) technology. The MLT technology, spacing differently the four-lines projected on the surface, allows for better management of problems that often affect other devices, such as holes, occlusions, or a consistently and rapidly changing depth range.

The laser scanner acquires the data in two different modes corresponding to two different baselines: *Macro* mode and *Wide* mode. Some constraints on the distance between the object and the scanner are given for each mode (Table 1). Activating the HD feature, it is possible to increase the point cloud density up to 4X. The choice of the mode depends on the object's size and the desired output accuracy. The scanner mounts a couple of twin arrays of four Class 1M 10-mW solid-state lasers with custom optics at a 650-nm wavelength. The scanner also features twin 3 MP CMOS RGB image sensors, and built-in white light texture illuminators [12].

## 3. Parameters Used for Characterizing the Texture of the Road Pavement

The roughness parameters of road pavement can be grouped into three classes. The first class takes into account “geometric” parameters related to the morphology of the sample analyzed. The second class includes the performance indicators, which are also intimately connected with the concept of skid resistance, analyzing the aspects of interaction between the tire and the road surface. The performance indicators are, in general, related to different conditions of measurement (speed of sliding, state of the surfaces, angle between the directions of motion and the tire, *etc.*), each with a specific corresponding measurement technique, often regulated by national (CNR, UNI) or international (EN, ISO)standards.

Among the tests commonly used to provide the most significant results are the Mean Texture Depth test (MTD, UNI EN 13036-1) which calculates the ratio between the volume of a material (sand or glass beads) and its footprint area after a coating is manual applied to the pavement, and the Pendulum test (PTV, Pendulum Test Value, UNI EN 13036-4) used for the measurement of the slip/skid resistance of a surface.

When compared with the methodology proposed, such empirical tests describe the texture in an indirect way, and present a quite high level of uncertainty in terms of results repeatability (just think, to the influence of the operator during the application of the sand or during the positioning of pendulum).

This work does not intend to use these tests as parameters for comparison, because they don't provide a direct measure of the texture.

The third class is composed by a series of statistical parameters, which are also related to the geometry of the profile: considering the high amount of data collected on a single sample and the possibility to assign them a physical meaning, the introduction of these parameters is highly justified for the more thorough study of road surfaces.

From the scientific literature and the standard rules and regulations, a set of parameters considered significant to characterize the texture [13,14] have been identified and selected. These parameters are shown in Table 2 and are divided into the above described classes.

**Figure 4.**
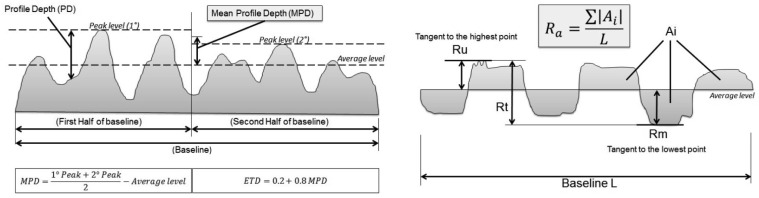
Descriptors detected by the use of profile meters.

The geometric parameters are derived from the characterization of the surface texture of the road pavement with the use of profile meters (UNI EN ISO 13473-1, ISO 13,473 to 2.3). In particular, the Mean Profile Depth (MPD) is defined as the difference between the arithmetic mean of two peaks and the mean level on a 100 mm baseline (UNI 13473-1:2004) (Figure 4). Once the MPD is obtained it is also possible to calculate the ETD (Estimated Texture Depth).

Other parameters in addition to the MPD are (Figure 4):

- average roughness (Ra): the average value of (absolute) deviations with reference to mean profile line;
- peak to valley height (Rt): the maximum vertical distance between the highest peak value and the lowest profile valley;
- leveling depth (Ru): the depth resulting from the distance between an average line and a straight line tangential to the profile peak;
- mean depth (Rm): the distance between an average line and a parallel line tangential to the most accentuated cavity, which is the lowest point.

**Figure 5.**
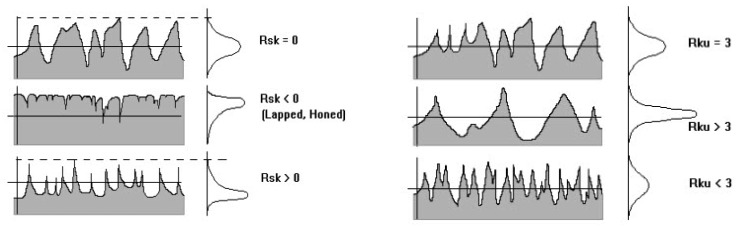
Physical meaning of the parameters Rsk and Rku.

The statistical parameters described in Table 2, variance (VAR), root mean square (Rms), asymmetry of the profile (Rsk) and kurtosis, which is an index of flattening of the profile (Rku), are defined in ISO 13473-2 standards (Figure 5).

The experimentation conducted in this work arises from the consideration that some of the commonly adopted methods have limitations derived by their complexity, costs, representativeness of the studied parameters and the limited reproducibility.

The current methodologies that are commonly employed in the road field for the determination of the geometric parameters in fact refer to measurements obtained using profile meters, which despite their high accuracy, present the disadvantage of providing point values and are heavily subject to the presence of singularities.

The research aim is to extend the texture reading, respect the traditional two-dimensional methods based on profiles, utilizing three-dimensional analysis, which is based on the results of high-resolution laser scans, and comparing the traditional performance indicators used to measure friction with the new indicators that are proposed.

In order to perform a technical assessment, with reference to the performance of the laser scanner in the texture characterization, it is necessary to compare its characteristics with those of the current instruments used in the road field.

The specifications of profile meters are given by the standards in terms of accuracy and the sampling rate; in general these parameters are more restrictive than those achievable with the instrumentation used in this work, but allow only a reading of one-way texture.

Therefore, the purpose of this work is to assess the comparability of the data and to extend the data analysis to a larger reference area to mitigate the uncertainty due to the choice of the profile. For this reason, in addition to the traditional parameters, others borrowed from the metrology are able to exploit the three-dimensional character of the acquired data for texture characterization.

## 4. Survey of Road Pavements with Laser Scanner

**Figure 6.**
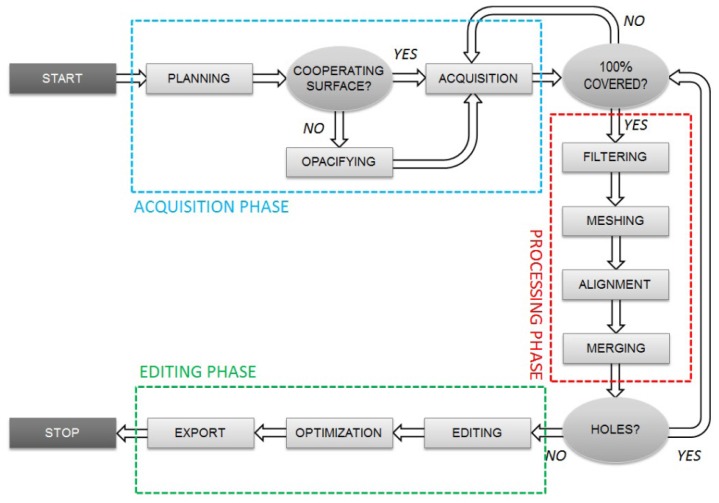
Flowchart of a 3D laser scanner survey.

The laser scanner survey workflow and the subsequent 3D model generation are usually performed by a succession of operations. This begins with a planning phase, where some key decisions are taken, such as the type of instrumentation that should be used in relation to the purposes for which the model is generated, passing through the data acquisition and finally ending with the post-processing operations. Figure 6 illustrates the main steps and phases, which constitute the entire workflow for a laser scanner survey.

The functioning behavior of laser scanners often deviates from the ideal conditions; there are a number of error sources that negatively impact the triangulation lasers performance by causing reduced accuracy, or even complete failure. These include, for example, adverse environmental illumination, object shape or object color, resulting in a lack of data, or a characterization of the surface that is very different from that which could be achieved under the ideal technical specifications of the instrument [15,16]. Problems can consist of a geometrical variation between the correct position of individual points and the point cloud acquired, or a radiometric type variation that leads to an altered colors texture. For these reasons, during the acquisition phase it is necessary to take all the precautions that make the surface optically cooperative and able to return the three-dimensional information as correctly as possible.

For the purposes of this application, which is functional to geometric and statistical analysis on the acquired surface, the 3D model is not the final product of the work. Hence the terminal phases of the process (editing and optimization), which generally are of great importance in three-dimensional modeling, have been subject to very mild or no intervention.

In the presented case study, in order to optimize the result of scanning for roads applications, a set of parameters have been preset by software: some dependent and correlated with the type of object, such as the color surface (glossy, matte, *etc.*), and others related to the mechanical performance of the instrument (operating mode, number of scans, quality of scans, *etc.*). The acquisition has been conducted in both modes, *Macro* and *Wide*, by setting the instrument in accordance with the highest quality available, and this has led to an acquisition time of about 120 seconds for each scan.

### 4.1. Data Acquisition

**Figure 7.**
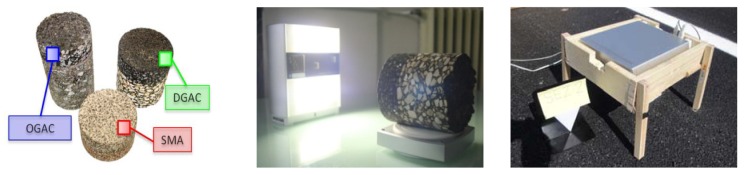
From left: road specimens, laboratory acquisition and on-site acquisition.

The objects that have been scanned (Figure 7) include a series of road pavement specimens cored on site and some others reproduced in the laboratory, simulating the installation conditions. The cylindrical specimens used in laboratory have a diameter of about 15 cm, and are representative of three different types of road pavement: Dense Graded Asphalt Concrete (DGAC), Open Graded Asphalt Concrete (OGAC) and Splitt Mastix Asphalt (SMA).

Some acquisition have also been performed directly on the paving site, in order to assess the applicability of this technique in all operating conditions, considering also the possibility to get repeated measures for future monitoring purposes. For the tests performed on-site, the pavement can be classified as SMA.

Working in *Wide* mode, a single scan is sufficient in laboratory to acquire the entire upper surface of a cylindrical specimen, while in *Macro* mode, having a smaller field of view, more scans are needed to cover the surface: three to six acquisitions were performed, depending on the size of the overlap area between the scans, the type of material, and the actual size of the specimen.

Considering on-site acquisitions, the aim of the work is also to assess the capacity and potential of a technique such as laser scanning to monitoring the variations of the pavement texture in a certain period, and to assess the roughness degradation due to the transit of vehicles over the time. The geometric shape of the measured area is not constrained, as in the case of the circular specimens, and for this reason the rectangular region provided by the scanner has been considered sufficient and easy to manage. Diurnal surveys have been carried out, with good weather conditions including sunlight and dry pavement. The surveys have been conducted taking advantage of the traffic closure for carrying out road consolidations. Some scan positions have been chosen aligned along the most probable trajectory of the vehicles, allowing future studies of the pavement texture variations due to the effects of acceleration and braking.

### 4.2. Data Processing

**Figure 8.**
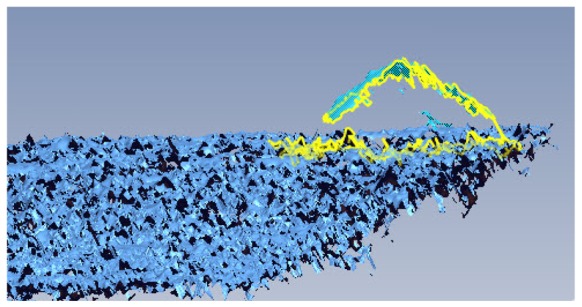
Removing the outliers for the 3D models.

Once the acquisition phase is complete, having taken care to cover the entire object surface, the scans are subjected to a preliminary filtering step. It is useful at this stage, also to reduce the following computational time, to eliminate all those parts that do not belong to the object. The filtering of a raw unfiltered dataset is a task that must always be done. Omitting this step could lead to topological errors when creating the mesh or during the alignment procedures. The parts that must be filtered include portions of the frame that support the specimens during the acquisition in laboratory, or any outliers (Figure 8) that might affect the results. Outliers also frequently affect the acquisitions on-site, as they consist of any grit conglomerate that is detached from the pavement, foliage, or other type of inert object not perfectly removed before scanning. Once the meshes are obtained, the *.*obj* file format is commonly used to export them; this file format contains information about the position of each mesh vertex, the direction of the normal of its faces, and information relating to the texture.

**Figure 9.**
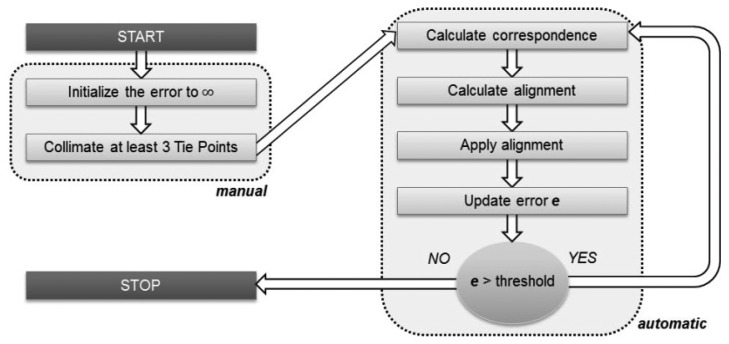
Alignment: Flowchart of the ICP algorithm.

When surveying the specimens, a single scan acquisition often is not enough to capture the entire surface, and in these cases multiple scans must be combined together. Each mesh, oriented with its own scanner reference system, requires a relative repositioning in order to reconstruct the original object geometry. Usually, after the approximate 3D roto-translation matrix is calculated in a preliminary phase, where at least three tie-points in two scans are selected, a second step with automatic ICP (Iterative Closest Point) procedures are involved. ICP algorithms perform a fine alignment by minimizing the difference between two point clouds according to some criteria (Figure 9).

**Figure 10.**
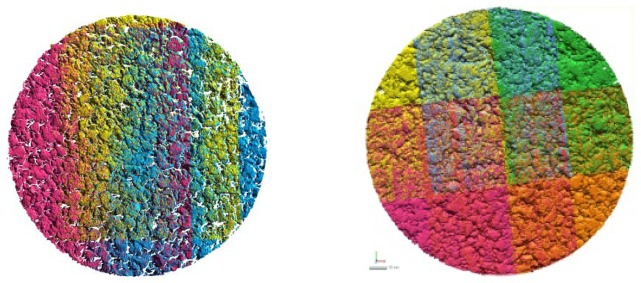
The image represents two different configuration of multiple scans merged together (*Macro* mode). According to the size and the diameter of the specimen, the number of scans can vary from 3 up to 6.

Once all the meshes are aligned in the same reference system, they have to be merged together in order to create a unique surface. Figure 10 represent with different colors each scan that have been acquired.

The merging phase introduces an additional level of data filtering. Usually performed by automatic procedures, this step is useful to eliminate topological errors (discrepancies between the meshes), to reduce redundant points, and to avoid the presence of gaps and noise on the final surface. These defects can be classified as:

- Non-manifold condition: when poly-faces share three or more poly-edges;
- Crossing condition: some edges intersect each other;
- Redundancy condition;
- Reversed normal in adjacent poly-faces.

Editing the model involves correcting all of these types of imperfections, keeping as much of the original geometry as possible. For the specimens analyzed, the result of these operations has been a 40–45% reduction in the starting number of points (approximately 1.5 to 2.5 million), thereby generating a smaller and more evenly distributed point cloud. The final product consists of a DSM (Digital Surface Model) structured as a mesh that is representative of the specimen surface.

**Figure 11.**
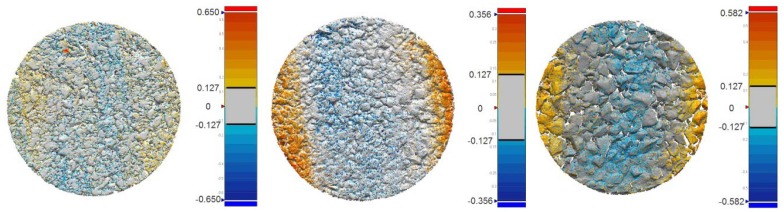
Deviations (mm) between the meshes obtained by *Macro* and *Wide* mode scans for the three types of pavement analyzed (DGAC, SMA, OGAC).

**Figure 12.**
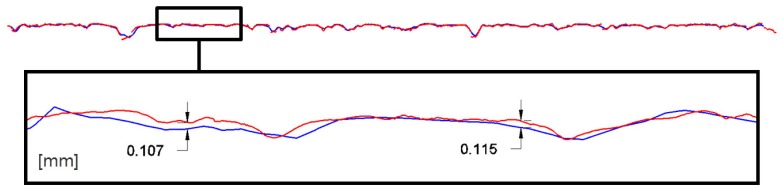
Deviations (mm) on a section profile between the *Macro* mode (red) and the *Wide* mode (blue) scans.

A first consideration regards the choice between the two acquisition modes, *Macro* and *Wide*. A comparison of execution speed and accuracy of the results has been carried out. The meshes obtained for the same area in both *Macro* and *Wide* modes have been overlapped to analyze the discrepancies between them. The test has been carried out for all the three kinds of specimens (DGAC, SMA and OGAC). Once the two meshes were aligned, the *Wide* one was subtracted from the *Macro* one. The results are shown graphically in Figure 11; it should be noted that for nearly all of the area considered, the deviation between the two surfaces is contained within a tolerance range that is assumed to be equal to 127 microns (1σ), a value corresponding to the accuracy declared by the manufacturer. The same data can also be obtained from the analysis of individual sections, one of which is shown in the subsequent Figure 12.

From this test it is possible to deduce that when operating on circular specimens, the *Wide* mode makes it possible to represent the entire surface with a single scan, with a fewer number of “holes” (dropout), therefore requiring less filtering operations by avoiding the merging step. On the other hand, the surface is smoother with respect to the *Macro* mode, which requires a higher number of scans and a consequently longer acquisition time. This depends on the point-to-point minimum distance, two times larger than in *Macro* mode; this space does not allow for a perfect micro roughness reading. Despite this finding, the *Wide* mode may still have a sufficient accuracy for expeditious field operations. In the following part of the paper, the authors use the *Macro* mode as the reference method for calculations on indicators of texture for both laboratory and on-site surveys.

## 5. Parameters Extraction from Laser Data

**Figures 13.**
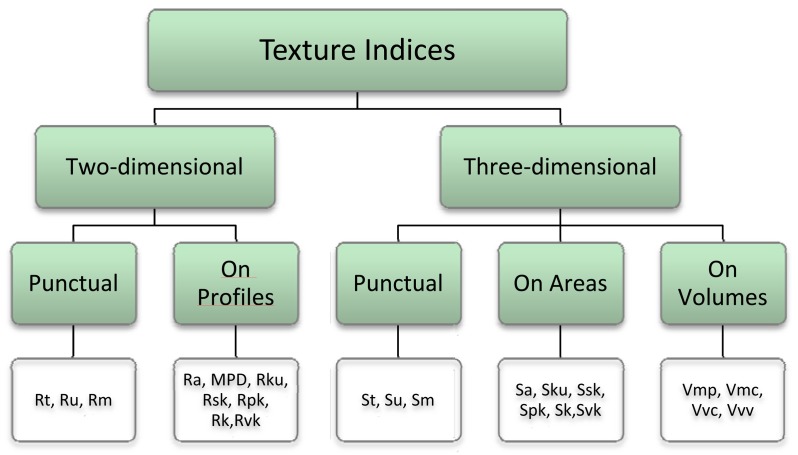
Classification of the texture parameters obtainable by a laser scanning.

The use of the laser scanner for studying the pavement texture allows for both the reproduction of the parameters measured by profile meters and the possibility to extend this methodology to a three-dimensional context, as shown in Figure 13.

The concepts expressed by the indices described in Paragraph 3 may in fact be extended not only to profiles, but also to the whole sample, in order to ensure a more representative description of the specimen cored on site. When working in three-dimensions, additional parameters can be considered as a way to retrieve the values of the material and voids, including studies on areas, projections on reference plans, and volumes referring the average-plane or a chosen secant one.

The geometric and statistical parameters previously expressed can thus be expanded and become representative of the entire area.

### 5.1. Two-Dimensional Parameters

In order to compare the “profiles” and the “areal” approaches, considering the small size of the cylindrical specimens and the need to have a sufficient number of measures to assess the results of the profile meter simulation, radial sections have been carried out.

Each mesh characterizing the specimen surface has been sectioned into eight normal planes, rotated with 22.5° separating them. With regards instead to the survey performed on-site, the rectangular footprint of the scanned area made the profile extraction easier because parallel planes have been adopted. From a profile metric analysis of the specimen scanned in laboratory and *in situ*, it is possible to obtain the two-dimensional parameters mentioned in Paragraph 3. Table 3 shows the data referring to the specimens scanned. The punctual parameters, even if they come from multiple profiles, and the MPD, as an average of two punctual values on the section, are strictly dependent on the random choice of the profile. This choice can influence the data since the presence of singularities in the sample (peaks or valleys accentuated) leads to high values of the parameters that are not representative of the entire surface. Even for the remaining parameters, the analysis of a single profile is not recommended, as an average of multiple sections leads to a greater representativeness of the data.

The more representative parameter of the roughness is Ra (area/length), since in a comparison of the single profiles it has been found to have minor deviations within the single scan. The parameters Rsk, representative of a greater presence of peaks or valleys in the sample, and Rku, varying from profile to profile, increase representativeness if they are averaged between a high number of sections. Reading the averaged profile meter parameters, in which it appears that the sample OGAC, typically rougher, has the highest indicators compared to the others, the different texture of the three samples is evident.

### 5.2. 3D Texture Parameters

Most of the 2D parameters defined in the ISO 4287 have a mathematical expression that can easily be extended to 3D. Table 4 reports an example of this extension for the previous two-dimensional parameters. Hereafter all indices in the 3D analysis start with the prefix “S” (meaning “surface”), replacing the “R” which was related to the 2D.

Punctual parameters retrieved from the entire area present values significantly higher than the counterparts retrieved from profiles. In addition, considering surveys on site, they vary a lot also within the same pavement. These problems underline the finding that the punctual parameters are not very representative, since a small singularity can push the values out of a reasonable range.

Parameters retrieved using the entire surface increase the representativeness of the profilometric ones, because using the area is like calculating the mean of an infinite series of profiles. The parameter Sa is very similar to its correspondent Ra, but is easier to calculate, proving to be a valid alternative to the latter. Ssk and Sku present significant differences with the analogues Rsk and Rku, but can be considered more reliable since the number of point is bigger. One of the more interesting applications involving 3D surfaces concerns the possibility to perform volumetric investigations, for example flooding simulations in the event of rain.

**Figure 14.**
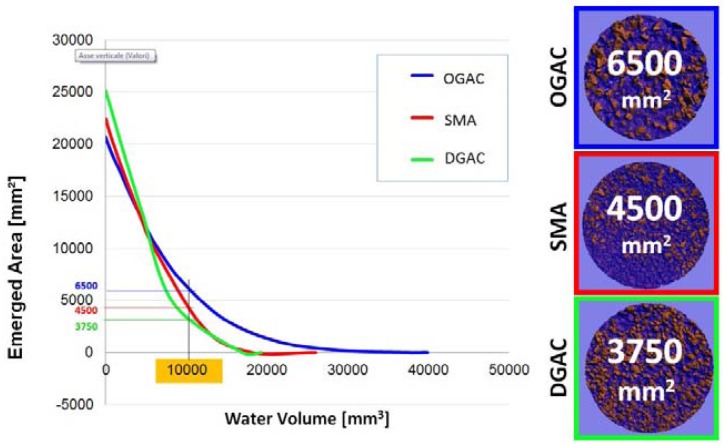
Analysis of Emerged Area *vs.* Water Volume: comparison between three different types of pavements. Below is shown an example of emerged area for a water volume of 10,000 mm^3^.

Assuming the water level as a uniform plane intersecting the roughness, it is possible to evaluate by a software procedure the emerged surface of asphalt when the volume of water increases. For this purpose, the secant plane was varied with Z steps equal to 0.5 mm in order to simulate the rain with a sufficient consideration to the filling of pavement. Figure 14 shows some significant moments of flooding simulation, where 0 represents the mean plane interpolating the point cloud. The numerical simulation was performed in conditions of “static” flooding, without considering some boundary conditions which may influence the traction in wet conditions, such as the draining effect of the outflow channels in the pavement, and the removal of a portion of water by the tires.

The previous considerations can be used as a start point for further studies, such as “void volume analysis” or performance assessments on the part of the pavement that has an active role in tire-road interaction.

In the analysis of pavement texture, new parameters can be introduced that are not commonly used for road and infrastructural applications. They are widely used in metrology for industrial applications, since are defined in the ISO standards; actually, the mechanical engineering industry, and in particular the automotive industry, has tried to find ways of optimizing parameters and filtering methods in order to make them more effective and to improve their correlation with functional phenomena [17].

The ISO 13565 standard introduces the Abbott curve, which represents mathematically the cumulative probability density function of the surface profile's height and can be calculated by integrating the profile trace.

**Figure 15.**
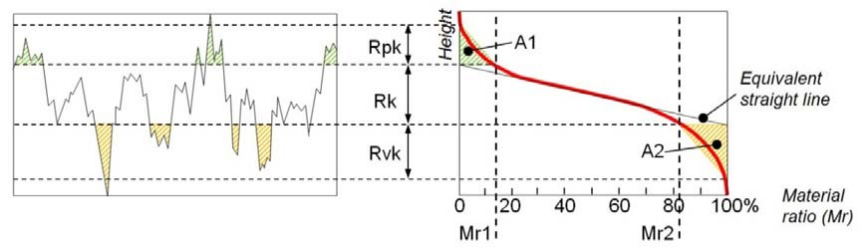
Abbot curve for a single profile and the parameters Rk, Rpk and Rvk.

Using the Abbott curve, a graphical study can be performed in order to retrieve functional parameters characterizing the roughness profile. According to Figure 15, for one profile it is possible to extrapolate the parameters Rk, Rpk, and Rvk; respectively representing the core roughness profile excluding the protruding peaks and the deep valleys, the average height of the protruding peaks above the core profile, and the average depth of the profile valleys projecting through the core profile.

The parameters Rpk and Rvk are each calculated as the height of the right-angle triangle, which is constructed to have the same area as the “peak area” (A1) or “valley area” (A2), respectively. Mr1 and Mr2 represent the percentage limits of the core roughness profile.

**Figure 16.**
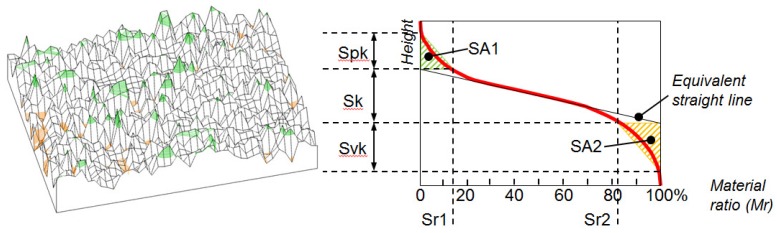
Abbot curve for one surface and parameters Sk, Spk, Svk.

Taking the previous concepts from one profile to one surface, the Abbott curve for the scanned area can be calculated. As defined in the standard ISO 25178 and shown in Figure 16, the 3D counterparts Sk, Spk and Svk, and SA1, SA2, Sr1 and Sr2, are calculated in the same way with respect to the Abbott curve, also computed on the entire surface.

Working with a 3D surface, we can go one step further and move to an evolution of the indices, called volume parameters, calculated with respect to the Abbott curve.

**Figure 17.**
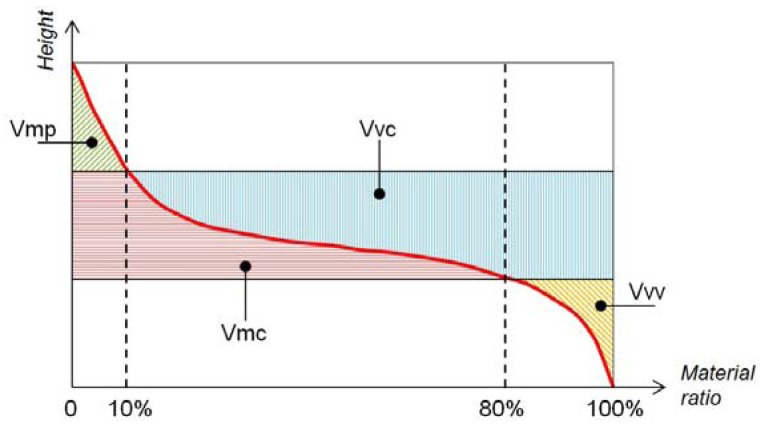
Abbott curve and volumetric parameters.

The parameters are defined with respect to two bearing ratio thresholds, set by default to 10% and 80%. Two material volume and two void volume parameters are defined as shown in Figure 17: Vmp (peak material volume), Vmc (core material volume), Vvc (core void volume), and Vvv (valley void volume).

**Figure 18.**
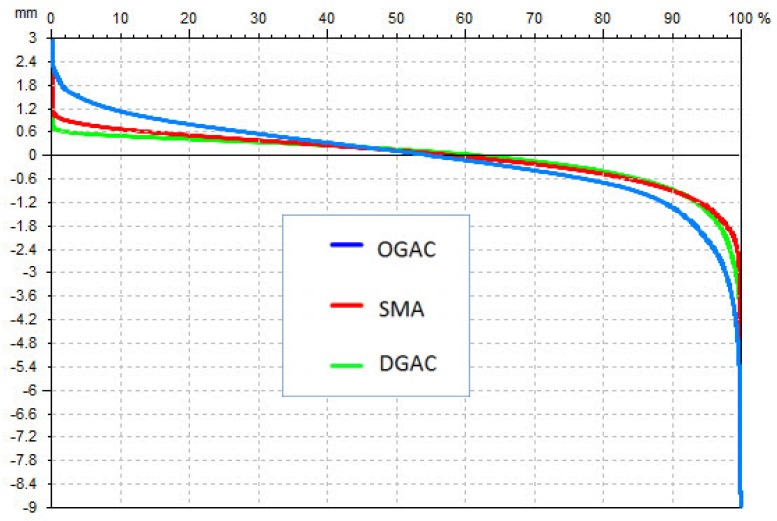
Abbott curve for the specimens DGAC, OGAC and SMA.

For the three types of pavements investigated, Table 5 shows a comparison between the parameters calculated as the mean of eight profiles, with the ones retrieved by the surface analysis. Figure 18 shows the three Abbott curves, where a more “horizontal” curve, like the green one belonging to the DGAC, means pavement is more compact.

The results from the 3D analysis evidence a correspondence between the values of the roughness parameters, and the intrinsic characteristics of the type of pavements investigated. Matching expectations, the DGAC came up as the more compact pavement, and the OGAC as the more rugged.

This trend is not always confirmed by the 2D parameters, where the DGAC and the SMA sometimes fail to meet expectations. Again this finding underlines how a 3D investigation produces more reliable results than the ones performed on profiles.

Furthermore, the volume parameters shown in Table 6 are coherent with the different types of pavement, and are useful because it is possible to estimate the void volumes and material volumes of the core roughness profile. Currently these parameters are applied to studies related to the comparison of different materials, and in relation to the pavement performance on site.

## 6. Conclusions

The possibility that texture information can be obtained from three-dimensional data involves a series of advantages in monitoring the paving quality and in the characterization of the traditionally-used indicators.

In particular, it is possible to detect texture characteristics with high accuracy and reliability of the results. Three-dimensional parameters are representative of a portion of the road paving, with a high increment of data in respect to single profiles, and therefore are less affected by presence of singularities.

The use of a laser scanner allows the calculation of new texture indicators, that, referring to surfaces and volumes, permit a more stable and complete texture characterization, providing further possibilities of evaluating the pavement surface performances.

The proposed approach can constitute a useful method for fast surveys, both on site and in laboratory, to be offered together with, or in place of, traditional surveys, which usually are slower and less precise, e.g., the HS (Height of Sand) test.

Possible further applications of these indicators can be developed for the study of the tire-road contact, for the complete evaluation—*in situ* or by means of core sampling—of the pavement condition, for monitoring the useful life and for the evaluation of the uniformity of the paving. The 3D pavement surface model could be used to study in a “virtual environment” the tire-pavement interaction by analytical methods and related to the noise and the vibration produced by traffic.

With the creation of appropriate pavement databases, it is furthermore possible to suggest a CE pavement marking, as already done for tires (EC no 1222/2009), certifying the class and the textural roughness in post-paving.

## Figures and Tables

**Table 1.** Specifications of the Next Engine^®^ laser scanner, as given by the producer.

**Dimensions**	224 × 91 × 277 mm	

	**Macro Mode**	**Wide Mode**

**Field of View**	13 × 10 cm	35 × 25 cm
**Working distance**	18 cm	40 cm
**Accuracy**	±127 μm	±381 μm
**Resolution**	200 DPI	75 DPI
**Texture density**	400 DPI	150 DPI
**Points/sec**	50,000	50,000

**Table 2.** Texture indicators.

**Indicator**	**M.u.**	**Description**	**Class**

**MPD**	[L]	Mean profile depth	Geometrical
**Ra**	[L]	Average roughness	
**Ru**	[L]	Levelling depth	
**Rm**	[L]	Mean depth	
**Rp**	[L]	Surface roughness depth	
**Rt**	[L]	Peak to valley height	

**VAR**	[L^2^]	Variance	Statistical
**Rms**	[L]	Average quadratic deviation	
**Rsk**		Skewness	
**Rku**		Kurtosis	

**Table 3.** Two-dimensional parameters.

**Two-dimensional Profilometric Parameters**				

***Profilometric***				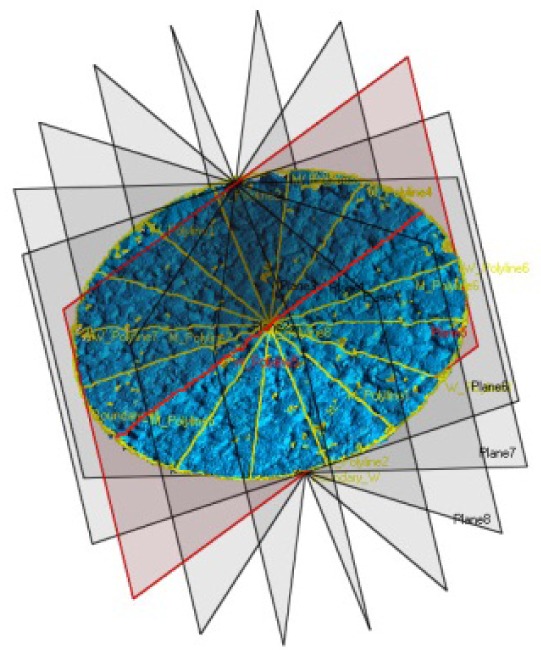

*indicator*	**DGAC**	**SMA**	**OGAC**	

**MPD** (mm)	0.65	0.97	1.54	
**Ra** (mm)	0.41	0.51	0.79	
**Rsk**	0.17	2.07	0.15	
**Rku**	2.27	−0.15	2.29	

***Punctual***				

**Rt** (mm)	3.32	3.84	9.01	
**Ru** (mm)	0.71	1.23	2.15	
**Rm** (mm)	−2.68	−2.61	−7.25	

**Table 4.** 3D parameters.

***3D parameters***				

***On Areas***				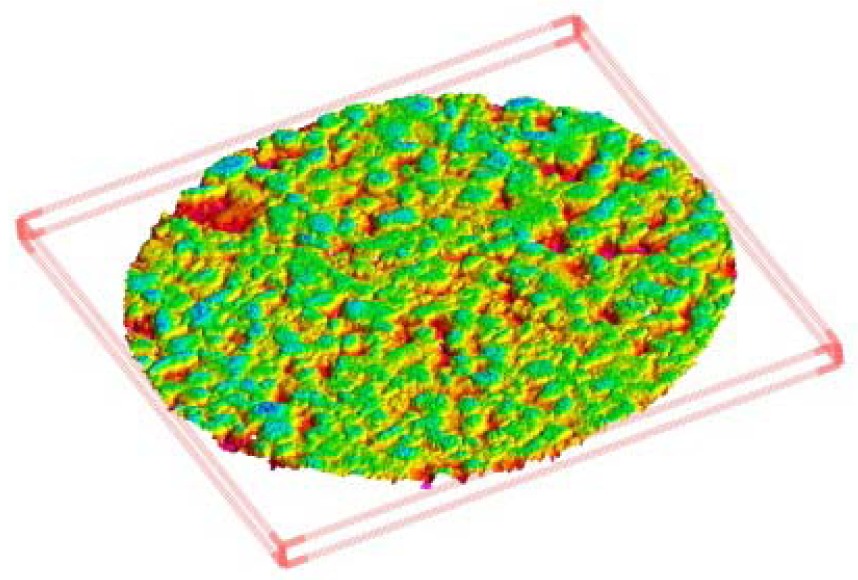

*indicator*	**DGAC**	**SMA**	**OGAC**	

**Sa**(mm)	0.46	0.50	0.80	
**Ssk**	2.24	−1.26	−1.4	
**Sku**	9.72	5.27	7.08	

***Punctual***				

**St** (mm)	6.13	6.26	11.22	
**Su** (mm)	1.02	1.44	2.58	
**Sm**(mm)	−5.11	−4.86	−8.63	

**Table 5.** Comparison between the 2D ISO 13565 and the 3D ISO 25178 parameters.

***Two-dimensional***				***Three-dimensional***			

**Indicator**	**DGAC**	**SMA**	**OGAC**	**DGAC**	**SMA**	**OGAC**	**Indicator**

**Rk** (mm)	0.69	1.02	1.43	1.04	1.74	2.81	**Sk** (mm)
**Rpk** (mm)	0.36	0.25	0.47	0.09	0.20	0.64	**Spk** (mm)
**Rvk** (mm)	1.12	0.76	1.58	1.53	1.69	2.68	**Svk** (mm)

**Table 6.** Volume parameters.

**Indicator**	**DGAC**	**SMA**	**OGAC**	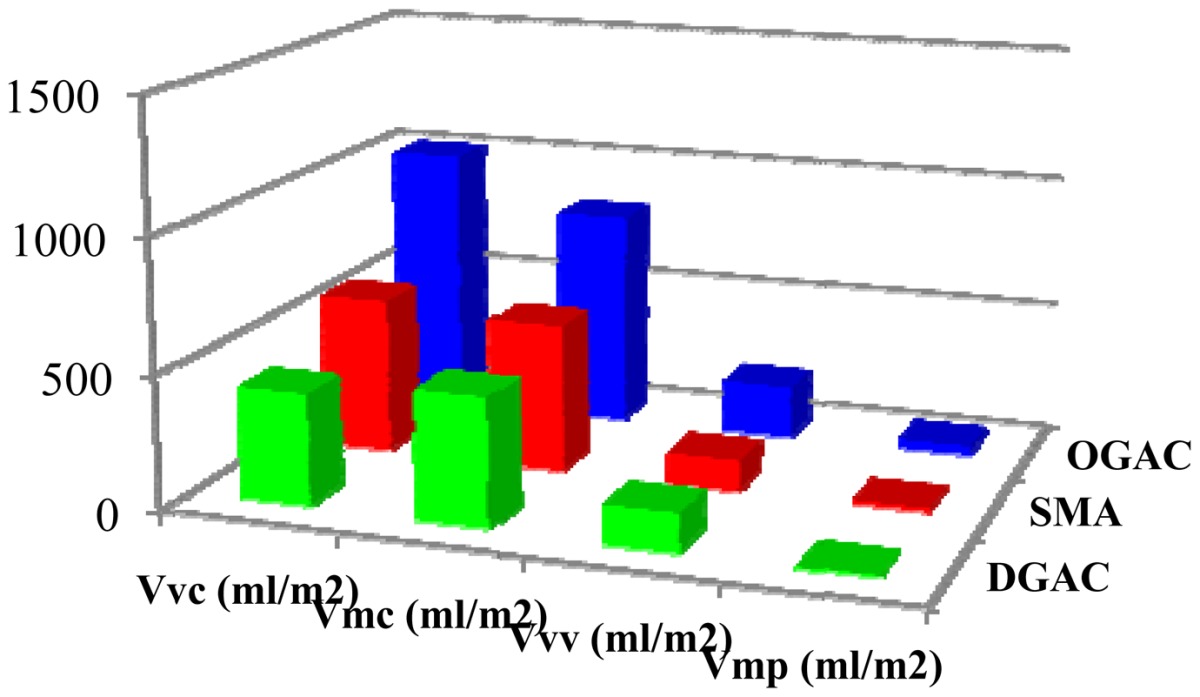

**Vmp** (mL/m^2^)	6.35	13.7	34.9	
**Vvc** (mL/m^2^)	419	587	1011	
**Vmc** (mL/m^2^)	480	557	816	
**Vvv** (mL/m^2^)	145	117	199	

## References

[1] PIARC World Road Association Report of the Committee on Surface Characteristics.

[2] Sandberg U., Descornet G. Road Surface Influence on Tyre/Road Noise—Parts I and II.

[3] Woodward D. From Vehicle/Surface Interaction to Quiet Surface Dressings.

[4] Henry J.J. (2000). Evaluation of Pavement Friction Characteristics.

[5] Flintsch G.W., De Leon E., McGhee K.K., Al-Qadi I.L. (2003). Pavement Surface Macro-Texture Measurement and Application.

[6] Simone A., Vignali V., Maglionico M., Bragalli C. Surface Run-Off: A Rainfall Simulator for Wash-Off Modellingand Road Safety Auditing under Different Rainfall Intensities.

[7] Choi Y. (2011). Review of Skid Resistance and Measurement Methods.

[8] Abbas A., Emin K., Azari H., Rasmussen R. (2007). Three-dimensional surface texture characterization of portland cement concrete pavements. Comput. Aided Civil Infrastr. Eng..

[9] Vilaça J., Fonseca J., Pinho A., Freitas E. (2010). 3D surface profile equipment for the characterization of the pavement texture—TexScan. Mechatronics.

[10] Blais F. (2004). A review of 20 years of range sensors development. J. Electron. Imaging.

[11] Sansoni G., Trebeschi M., Docchio F. (2009). State-of-the-art and applications of 3D imaging sensors in industry, cultural heritage, medicine, and criminal investigation. Sensors.

[12] Guidi G., Russo M., Magrassi G., Bordoni M. (2010). A performance evaluation of triangulation based range sensors. Sensors.

[13] Boscaino G., Praticò F.G., Vaiana R. Spectral Texture Indicators Significance in Relation to Flexible Pavements Surface Performance.

[14] Boscaino G., Praticò F.G., Vaiana R. Texture Indicators and Surface Performance Significance in Flexible Pavements—SURF2004.

[15] Guarnieri A., Vettore A., Zanette S. Analisi Della Risposta Di Un Laser Scanner Terrestre Al Variare Delle Caratteristiche Di RiflettivitÀ Dei Materiali.

[16] Guidi G., Remondino F., Morlando G., Del Mastio A., Uccheddu F., Pelagotti A. Performances Evaluation of a Low Cost Active Sensor for Cultural Heritage Documentation.

[17] Blateyron F. New 3D Parameters and Filtration Techniques for Surface Metrology.

